# Chitosan as a Biomaterial: Influence of Degree of Deacetylation on Its Physiochemical, Material and Biological Properties

**DOI:** 10.1371/journal.pone.0135153

**Published:** 2015-08-25

**Authors:** Leslie John Ray Foster, Sonia Ho, James Hook, Monica Basuki, Helder Marçal

**Affiliations:** 1 Bio/Polymer Research Group, School of Biotechnology and Biomolecular Sciences, University of New South Wales, Sydney, Australia; 2 NMR Facility, Mark Wainwright Analytical Centre, University of New South Wales, Sydney, Australia; Monash University, AUSTRALIA

## Abstract

Chitosan is a biomaterial with a range of current and potential biomedical applications. Manipulation of chitosan degree of deacetylation (DDA) to achieve specific properties appears feasible, but studies investigating its influence on properties are often contradictory. With a view to the potential of chitosan in the regeneration of nerve tissue, the influence of DDA on the growth and health of olfactory ensheathing cells (OECs) was investigated. There was a linear increase in OEC proliferation as the DDA increased from 72 to 85%. This correlated with linear increases in average surface roughness (0.62 to 0.78 μm) and crystallinity (4.3 to 10.1%) of the chitosan films. Mitochondrial activity and membrane integrity of OECs was significantly different for OECs cultivated on chitosan with DDAs below 75%, while those on films with DDAs up to 85% were similar to cells in asynchronous growth. Apoptotic indices and cell cycle analysis also suggested that chitosan films with DDAs below 75% were cytocompatible but induced cellular stress, while OECs grown on films fabricated from chitosan with DDAs above 75% showed no significant differences compared to those in asynchronous growth. Tensile strength and elongation to break varied with DDA from 32.3 to 45.3 MPa and 3.6 to 7.1% respectively. DDA had no significant influence on abiotic and biotic degradation profiles of the chitosan films which showed approximately 8 and 20% weight loss respectively. Finally, perceived patterns in property changes are subject to change based on potential variations in DDA analysis. NMR examination of the chitosan samples here revealed significant differences depending upon which peaks were selected for integration; 6 to 13% in DDA values within individual samples. Furthermore, differences between DDA values determined here and those reported by the commercial suppliers were significant and this may also be a source of concern when selecting commercial chitosans for biomaterial research.

## Introduction

Chitosan as a biomaterial elicits a negligible immune response following implantation, injection, topical application or ingestion in mammalian systems [1]. Chitosan also possesses attractive haemostatic properties and is reported to stimulate the host immune system against viral and bacterial infections [2,3]. Furthermore, a number of studies suggest that chitosan may promote wound healing in both soft and hard tissue [4–6]. Consequently, chitosan has approval for application as a biomaterial by the Food and Drug Administration (FDA) in the USA and regulatory bodies of other countries, and is currently used in a number of commercial products from haemostatic bandages to dietary aides [6–8].

**Fig 1:**
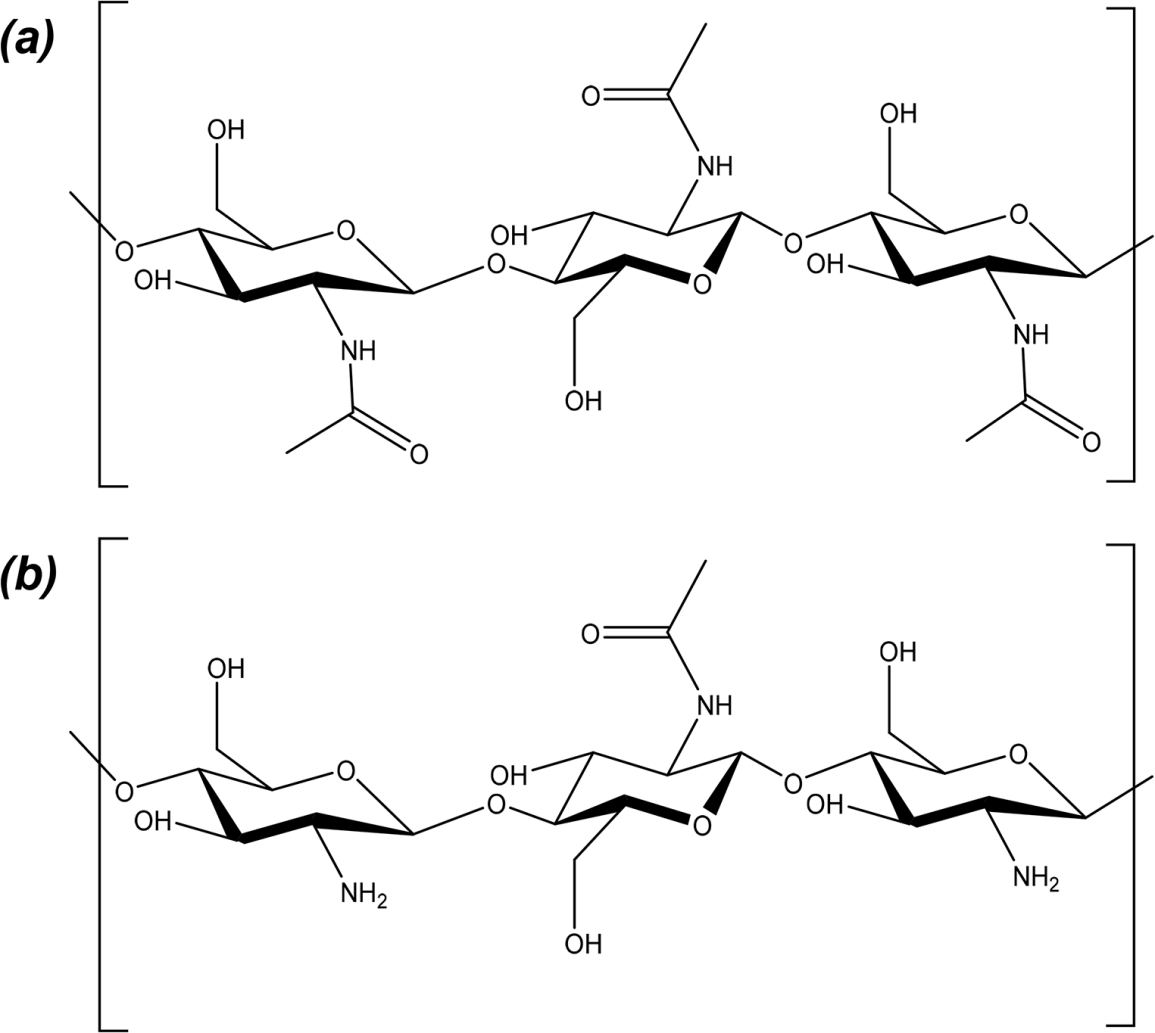
Chemical structure of chitin *(a)* and chitosan *(b)*.

Chitosan, a copolymer of D-glucoasmine and N-acetyl-D-glucosamine, is commonly derived through the N-deacetylation of chitin found in crustacean and insect carapaces, (Fig 1) [5]. With approximately 10^11^ tons of chitin produced per year from waste crustacean carapaces, chitosan is a cost effective biomaterial [4]. The degree of deacetylation (DDA) exhibited by chitosan can be controlled during a relatively aggressive alkaline hydrolysis process applied to the chitin, through a combination of exposure duration and temperature [9].

Irrespective of its DDA, chitosan is fully cytocompatible with a number of cell lineages [5]. However, cell adhesion and proliferation appear to be lineage dependent, thus Chatelet *et al*. reported that fibroblast attachment occurred with an inversely proportional relationship: the higher the DDA, the weaker the attachment, with no proliferation [5]. In contrast, the opposite is reported for keratinocytes and Schwann cells [10,11]. Similarly, a number of reports present conflicting trends in the influence of DDA on physiochemical and material properties [11].

In this study, we report on the influence of DDA on the physicochemical, material and biological properties of commercial chitosan samples with the view to using this attractive biomaterial for the development of nerve conduits. Current nerve repair is reliant upon autografts and allogeneic grafts [12]. However issues of rejection and limited supply of donor tissue drives research into biomaterials for the development of nerve guides or conduits. A nerve conduit should possess sufficient strength and flexibility to support nerve regeneration while withstanding compression by body tissue. After implantation, the conduit should degrade as the nerve regenerates. Nerve conduits fabricated from type I collagen, polyglycolic acid (PGA), poly-DL-lactide-co-caprolactone (PLCL) and polyvinyl alcohol (PVA) are currently used clinically [13].

The influence of DDA on the adhesion and proliferation of Schwann cells has been reported, however, there are a number of disadvantages to using Schwann cells, including their interaction with astrocytes and sampling problems [14]. Olfactory ensheathing cells (OECs) are a type of glial cell, vital in promoting the regeneration of nascent neurons in the olfactory system [15]. In contrast to Schwann cells, OECs can be easily collected with minimal invasiveness from adults and show no interaction with astrocytes. Furthermore, OECs are the only type of cell that continuously ensheath axons from the peripheral (PNS) to central nervous system (CNS) [16].

The potential application of chitosan for nerve repair and engineering requires two cellular processes to occur concurrently. Cells should grow and populate the biomaterial scaffold, whereby the cells cycle through various growth phases classified as ‘resting’ (G0/G1), ‘DNA synthesis’ (S), ‘mitosis’ (M) and the gap between the S and M phases; ‘G2’. In response to biomaterials, cells can change from a non-proliferative to a proliferative state which is typified by the relative reduction in the G0/G1 phase. Concurrently, cells may differentiate into specific lineages to fulfil specific functions. Undifferentiated cells are programmed to progress through the cell cycle, and while mature neurons exit and cannot re-enter the cell cycle, Gage has suggested that undifferentiated neural cells can be influenced in tissues such as the olfactory epithelium [17]. In this study, the influence of chitosan DDA on OEC cycle progression is reported as an indicator of cell health. In this paper we investigate the potential of readily available, commercial chitosan to promote OEC growth and health, with a focus on the influence of the biomaterial’s DDA.

## Materials and Methods

### Materials and reagents

Chitosan samples S1, S2 and S3 (#448869, 448877 and 419419 respectively) were purchased from Sigma (Seven Hills, Australia), while medical grade chitosan samples T1, T2 and T3 (#T9462, T9011 and T8736 respectively respectively) were purchased from Thahira Chemical Corporation (Delhi, India). Nocodazole and Aphidicolin were also purchased from Sigma-Aldrich (Seven Hills, Australia). Glacial acetic acid was acquired from Univar, (Sydney, Australia), Trypsin, Dulbecco’s modified Eagle’s medium supplemented with 10% foetal bovine serum (DMEM-FBS), Pen-Strep antibiotic and Fungizone (Amphotericin B) were obtained from Gibco BRL Life Technology, (Grand Island, NY, USA). Cell-Titer 96 Aqueous One Solution Cell Proliferation Assay was purchased from Promega (Madison, USA). All chemicals used were of analytical grade.

### Determination of DDA

The degree of deacetylation (DDA) for each chitosan sample was determined using a Bruker Avance 500 ^1^H-NMR operating at 500.13 MHz with a 5mm Indirect Detection probe at 343K. (Mark Wainwright Analytical Centre, UNSW, Australia). The following parameters were used: A 9.25 μs 90° pulse; spectral width, 6 kHz; acquisition time, 2.73 s; recycle time 5 s. Solvent suppression of the residual HOD peak was achieved using a pre-saturation pulse prior to data acquisition. The raw free induction decays (FIDs) were line broadened (0.2 Hz) prior to Fourier transformation, phasing, base line correction and manual integration. DDAs were calculated as per a method adapted from Lavertu *et al*. using spectra of chitosan solutions (10mg in 1.96ml D_2_O/0.04ml DCl) with the following formulae [18]:

$$DDA\;\left(\%\right)=\left(\frac{H1D}{H1D+HAc/3}\right)\times 100$$ Eq 1

$$DDA\;\left(\%\right)=\left(1\;-\;\left(\frac{1}{3}\;HAc/\frac{1}{6}\;H26\right)\right)\times 100$$ Eq 2

$$DDA\left(\%\right)=\left(\frac{H1D}{H1D+H1A}\right)\times 100$$ Eq 3

Where *H1D* is the peak corresponding to proton of the deacetylated monomer H1; *HAc* is the peak corresponding to the 3 protons of the acetyl group; *H26* is the signal from protons on H2 to H6 of both monomers and *H1A* is the peak corresponding to the proton on the acetylated monomer H1.

### Fabrication of chitosan films

Chitosan solutions (9 mL, 2% w/v in analytical grade acetic acid, 2% v/v, pH 6.4) were poured into sterile, glass Petri dishes. Thin films (approx. 20μm) were fabricated within 48 hours by standing in a clean laminar flow hood and left to anneal for a further 12 days (25°C, rH 30%) [19].

### Film characterisation: crystallinity

X-ray diffraction (XRD) patterns of the chitosan films were recorded using a Philips X'Pert PRO Material Research Diffraction (MRD) X-ray diffractometer, (PANalytical, Almelo, The Netherlands) with a radiation wavelength of 1.5406 Å, 45 kV power and 40 mA tube current using Cu-Kα radiation. The relative intensities were recorded in a scattering range (2θ) of 4–40°, and a scan step size of 0.02°. The film crystallinities (X_c_) were calculated as per Eq 4:

$$Xc=\left(\frac{Fc}{Fc+Fa}\right)\;x\;100\;\%$$ Eq 4

Where *Fc* and *Fa* are the areas under the diffraction peaks of crystal and non-crystalline regions, respectively [20]. Means of at least 10 replicates were determined (*n* = > 10).

### Film characterisation: surface roughness

Surface microtopographies of dry chitosan films were observed using the reflection mode of a confocal scanning laser microscope (CLSM, Leica TCS SP Confocal DMIRB, Germany) with an excitation of 458 nm and emission wavelength of 440–470 nm. Multiple images were taken through the *z* plane (step size = 0.5 μm). The average surface roughness values (*R*_a_) were calculated from these images using ImageJ software (National Institutes of Health, USA) according to ISO 4298 (2000):

$$Ra=\frac{1}{L}\int_{0}^{L}|z|dx$$ Eq 5

Where *L* is the sampling length, *z* the plane and d*x* the variations of irregularities from the mean line. Ten images were taken and the average *R*_a_ and mean facet orientation (MFO) determined [21].

### Film characterisation: hydrophobicity

The surface hydrophobicity of the chitosan films was determined using the sessile drop technique; water droplets from a microsyringe were placed on the surface of chitosan films and angles measured using a contact angle meter (KSV Cam 200, Finland) [22]. Means of at least 10 samples were determined (*n* > 10).

### Film characterisation: material strength

Mechanical properties of chitosan films were determined using an Instron 2752–005 tensile testing apparatus (Instron, MA, USA). Film strips of chitosan (30 x 15 mm) were held between two pneumatic clamps separated at a distance of 25 mm and pulled apart with a constant cross head speed of 20 mm min^-1^. Tensile strength and elongation at break were calculated using BlueHills software (Instron, MA, USA) [23]. Films exhibiting a DDA of 85% were also incubated in deionised water at 25°C for 2 hours and their mechanical properties tested. Means of at least 10 samples were determined (*n* > 10).

### Film characterisation: degradation

Preweighed, sterilised chitosan samples (12 mm diameter) were placed into 2 mL Eppendorf tubes filled with sterile PBS (0.1 M, pH 7.4) containing penicillin (100 units mL^-1^), streptomycin (100 μg mL^-1^) and Fungizone (2.5 μg mL^-1^) then incubated (37°C, 150 rpm). At periodic intervals film samples were removed, filtered (2 μm pore size) with deionised water to capture any particulate pieces and dried in a vacuum oven (60°C, 48 h) then left to stand in a clean laminar flow hood for moisture equilibration (25°C, rH 25% 12 h). The samples were subsequently weighed and weight loss (WL) expressed as percentages of the initial [24]:

$$\text{WL}\;\left(\%\right)\;=\frac{\left(W_{f}-W_{i}\right)}{W_{i}}\times 100$$ Eq 6

Where *W*_*f*_ and *W*_*i*_ are the final and initial weights respectively. The experiment was simultaneously conducted with the addition of lysozyme to final concentration of 0.056 g L^-1^ [25]. Means of 5 replicates were determined (*n* = 5).

### Cell proliferation and morphology

Prior to cell culturing, chitosan films were sterilised using Gamma irradiation for 20 min at a dosage rate of 0.564 KGy/h [26]. Adherent Olfactory Ensheathing Cells (OECs) were cultivated in sterile T75 tissue flasks containing DMEM-FBS medium, (37°C, 5% CO_2_) and harvested at 80% confluence [26]. Populations of approximately 1 x 10^5^ cells mL^-1^ were used to inoculate sterile chitosan films (13 x 13 mm) and incubated (37°C, 5% CO_2_). Controls inoculated in the absence of the polymers were simultaneously conducted. After 5 days samples were rinsed twice with 10 mL of PBS containing 2 mL of trypsin (2.5% v/v) before further incubating for 2 mins (37°C, 5% CO_2_). Cell proliferation and viability were determined using a Tali Image-Base Cytometer. Triplicates per film, per time point were used for each proliferation assay (*n* = 3).

OECs attached to polymer films were visualised using SEM as per Ahmed *et al*. [27] Cells cultivated on the polymer films had their medium removed and the specimens subsequently rinsed with 1% PBS twice prior to fixing in 0.1 M PBS containing 2.5% glutaraldehyde solution (22°C, pH 7.4, 4 h). Films were then washed with PBS buffer for 3 cycles (5 mins each) and subsequently dehydrated stepwise in a series of ethanol washes (from 30, 50, 70, 80, 90 and 95 to 100%) for 10 mins at each concentration. Films in 100% ethanol were critical point dried using liquid CO_2_ then mounted on aluminium stubs and sputter coated with gold (2 mins, 20 mA, Emitech K550x, Hertfordshire, England). Samples were examined using a Hitachi S3400-N scanning electron microscope (Japan) at 15 kV and 30 mA.

### Mitochondrial activity and membrane integrity

OECs cultured in DMEM-FBS medium, were harvested by trypsinisation as per above and populations of approximately 3 x 10^3^ cells mL^-1^ used to inoculate 96 well plates coated with polymer films, cells cultivated in the absence of the biomaterials were used as controls. Plates were incubated (37°C, 5% CO_2_, 48 h) and mitochondrial activity and membrane integrity assayed. Mitochondrial function was assessed using an Cell Titer 96 Aqueous one cell proliferation assay, 30 μl of MTS (3-(4,5-dimethylthiazol-2-yl)-2,5-diphenyltetrazolium bromide) solution was added to each well prior to 4 h incubation as above. MTS concentrations were then measured at 490/690 nm using a micro-titre plate spectrophotometer (Spectra Max 3400, Molecule Device, USA).

Lactate dehydrogenase (LDH) release was used as a measure of membrane integrity in OEC populations as a consequence of their incubation with the chitosan films [28]. At 45 mins prior to the incubation endpoint, 10 μl samples of lysis solution were added to 5 of the wells and these served as positive controls. Plates were centrifuged (300 g, 5 min, 22°C) and supernatant samples (50 μl) transferred to each well of a sterile 96 well plate. LDH mixture (100 μl) was then added to each well before incubating in the dark (30 mins, 37°C, 5% CO_2_). LDH analysis was performed at 490/650 nm using the micro-titre plate spectrophotometer. Means of 5 replicates per sample were determined (*n* = 5).

### Cell cycle and apoptosis

OECs were cultured in DMEM-FBS medium (12 mL) in sterile glass tissue culture dishes coated with the chitosan films and incubated for 5 days (37°C, 5% CO_2_). Controls for DNA content were simultaneously conducted including: (1) cells in the absence of chitosan films, (2) cells in serum-free medium, and cells synchronised (48 h) in medium containing either 1μg mL^-1^ aphidicolin (3) or 2 μM nocodazole (4). Similar controls, but with the absence of (3) aphidicolin, were used in the determination of apoptotic indices associated with membrane externalisation of phosphotidylserine (PS) [29,30].

After 5 days incubation, OECs were trypsinised and excess FBS media used to neutralise the trypsin reaction. Cells were then washed with sterile PBS and fixed using ethanol (70%, -20°C, 12 h) before centrifuging (300 g, 5 min). Cell pellets were resuspended in propidium iodide (PI) staining solution (0.1% v/v Triton X-100, 0.2 mg mL^-1^ RNase, 20 μg mL^-1^ PI in PBS) and incubated in the dark (22°C, 30 min) before cell cycle analysis using a Tali Image-Base Cytometer (Invitrogen, USA). For apoptosis analysis, OEC samples were washed with PBS and suspended in Annexin binding buffer and Annexin-V Alexa Fluor 488 (Invitrogen, USA) prior to incubation in the dark (22°C, 30 min). Cells were then extracted through centrifugation (300 g, 5 min), suspended in Annexin binding buffer and Tali PI solution added before further incubation in the dark (22°C, 5 min). The Tali Image-Base Cytometer was then used to determine apoptotic indices [28,30]. Means of 3 replicates per sample were determined (*n* = 3).

### Statistical analysis

All data was obtained from 3 separately prepared films per chitosan sample and statistically evaluated using the one and two-256 way ANOVA analyses and Bonferroni post-test, where appropriate, with 95% significance.

## Results and Discussion

### Determination of DDA

**Fig 2:**
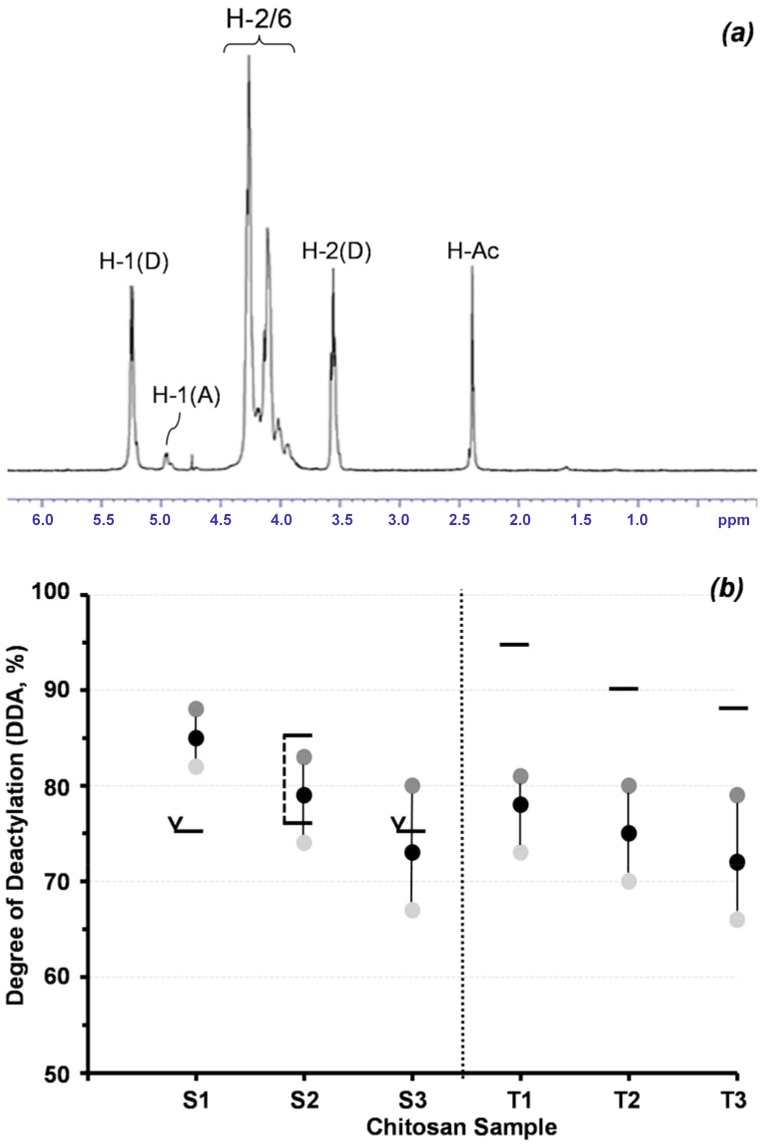
*(a)* ^1^H NMR spectrum of commercial chitosan sample S1 (10 mg i 1.96 ml D20/0.04 ml DCl) and *(b)* Variation in degree of acetylation of commercial chitosan samples as determined by NMR, as reported by supplier: (horizontal black line); range: (black corner); and (horizontal black line with corner arrow); greater than S1-S3: supplied by Sigma-Aldrich; T1-T3: supplied by Tahira Chemical Corp.).

DDA in commercial chitosan samples is often determined using rheology, however this is prone to error [31]. Similarly, peak integration through NMR can show different DDAs dependent upon sample preparation [18]. In the study here, three different peak integrations were used to accommodate for possible variations in preparation (Fig 2A). The variations in DDA for each sample, based on these integrations ranged from 6 to 13% with mean DDAs ranging from 72 to 85% (Fig 2B). Furthermore, the DDA values determined here using NMR differed significantly from those reported by the chitosan suppliers. While S2 and S3 purchased from Sigma Aldrich had DDA values within the ranges reported (75 to 85% for S2 and >75% for S3), a value of >75% for S1 was significantly less than the mean of 85% determined in this study. In contrast, the DDAs for chitosan samples purchased from Thahira Chemical Company showed values up to 18% lower than those provided (Fig 2B). Such significant variation in DDA could help explain contrasting trends in chitosan properties as reported by different authors [3,5,11,32]. In the study here, means of the 3 different NMR values for each sample were used.

### Film characterisation

**Fig 3:**
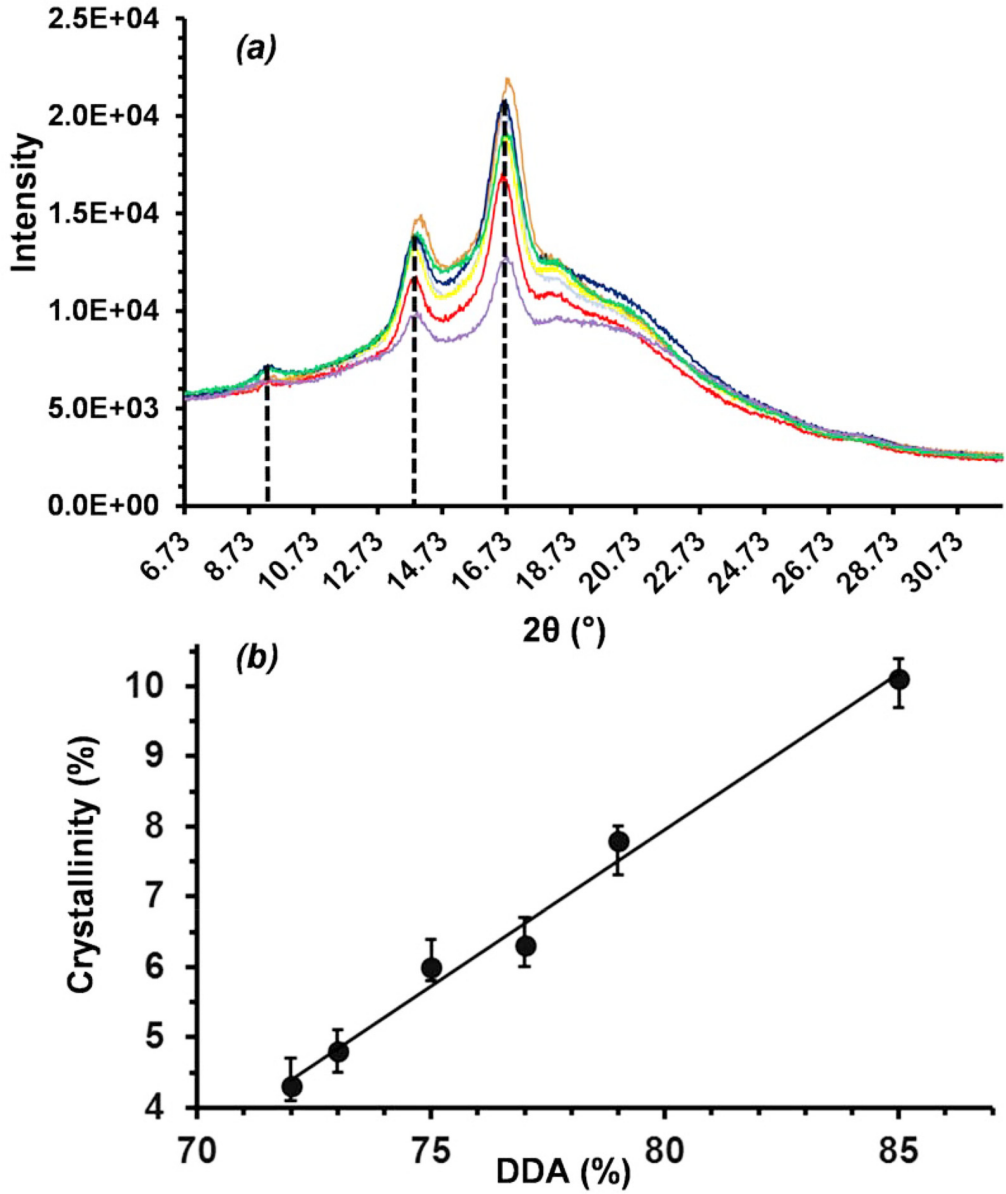
X-ray diffractograms of solvent cast films from different commercial chitosan samples *(a)* and corresponding changes in crystallinity *(b)*.

Changes in DDA appeared to have no significant effect on crystallisation behaviour with X-ray diffraction peaks at 10, 14 and 17° (2*θ*), consistent for each sample and in agreement with the literature (Fig 3A) [33,34]. However, relative peak intensities increased with DDA, corresponding to an increase in crystallinity. Fig 3B shows a linear increase (R^2^ = 0.9878) in crystallinity with increasing DDA from 4.3 ± 0.3 to 10.1 ± 0.3% for films fabricated from chitosan samples with 72 and 85% DDA respectively [34]. Crystallinity may also have been influenced by changes in the molecular weight of the samples; while all samples had reported molecular weights around 190 k, sample S1 was reported to have values as low as 50 k, while S3 up to 357 k. While acid hydrolysis can lead to depolymerisation and a subsequent reduction in molecular weight, minimal chain scission was anticipated in the study here given the weakly acidic chitosan solution (pH 6.4) and the relatively short film fabrication time (48 h). Furthermore, film fabrication techniques were standard and consistent for all samples, with the chitosan samples and their DDA the only variable.

**Fig 4:**
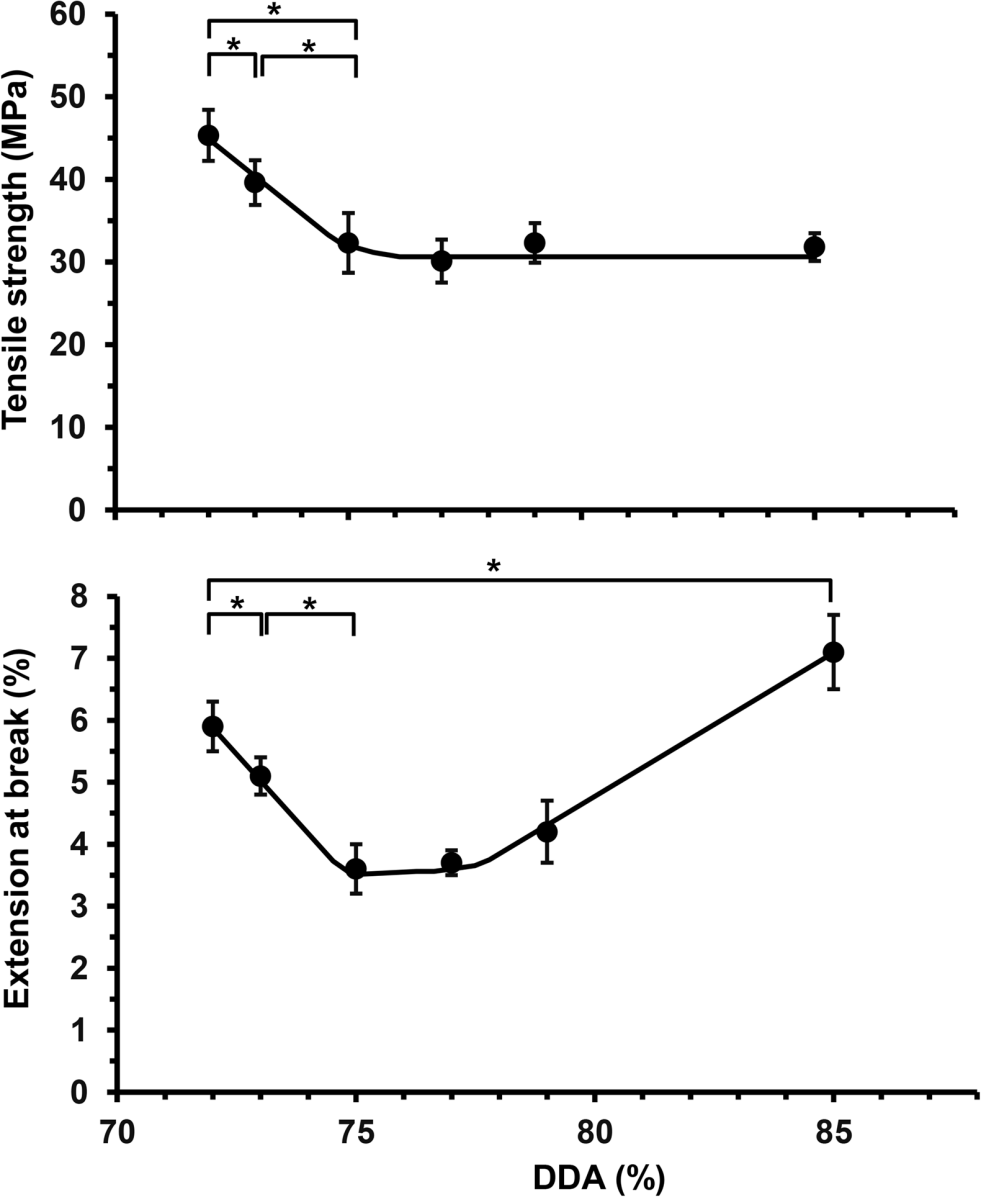
Variation in mechanical properties of solvent cast films fabricated from commercial chitosan samples with different DDAs, as measured by *(a)* tensile strength (MPa) and *(b)* elongation at break (%), (*Significant difference, P < 0.05, *n* = ≥ 10).

Crystallinity is known to influence tensile strength and there were significant differences in the mechanical properties of chitosan films fabricated through solvent casting from acetic acid. Increasing the DDA from 72 to 75% was accompanied by a significant reduction in tensile strength from 45.3 ± 3.1 to 32.3 ± 3.6 MPa. This initial decrease in tensile strength for was also matched by decreases in their elongation to break; films with a DDA of 72% possessed an elongation at break of 5.9 ± 0.4% which decreased to 3.6 ± 0.4% for films fabricated from chitosan with a DDA of 75%. However, chitosan films with DDAs 75 to 85% showed no significant differences in tensile strength but showed a gradual increase in elongation at break, such that films prepared from chitosan with a DDA of 85% showed an elongation at break of 7.1 ± 0.6% (Fig 4). At relatively low DDAs these results are consistent with Chatelet *et al*. who reported an increase in brittleness as the DDA increased [5]. At higher DDAs the mechanical performance was more consistent with that of Wenling *et al*. who correlated the inverse relationship between chitosan swelling and mechanical strength, as swelling increases, the mechanical strength decreases [11].

**Fig 5:**
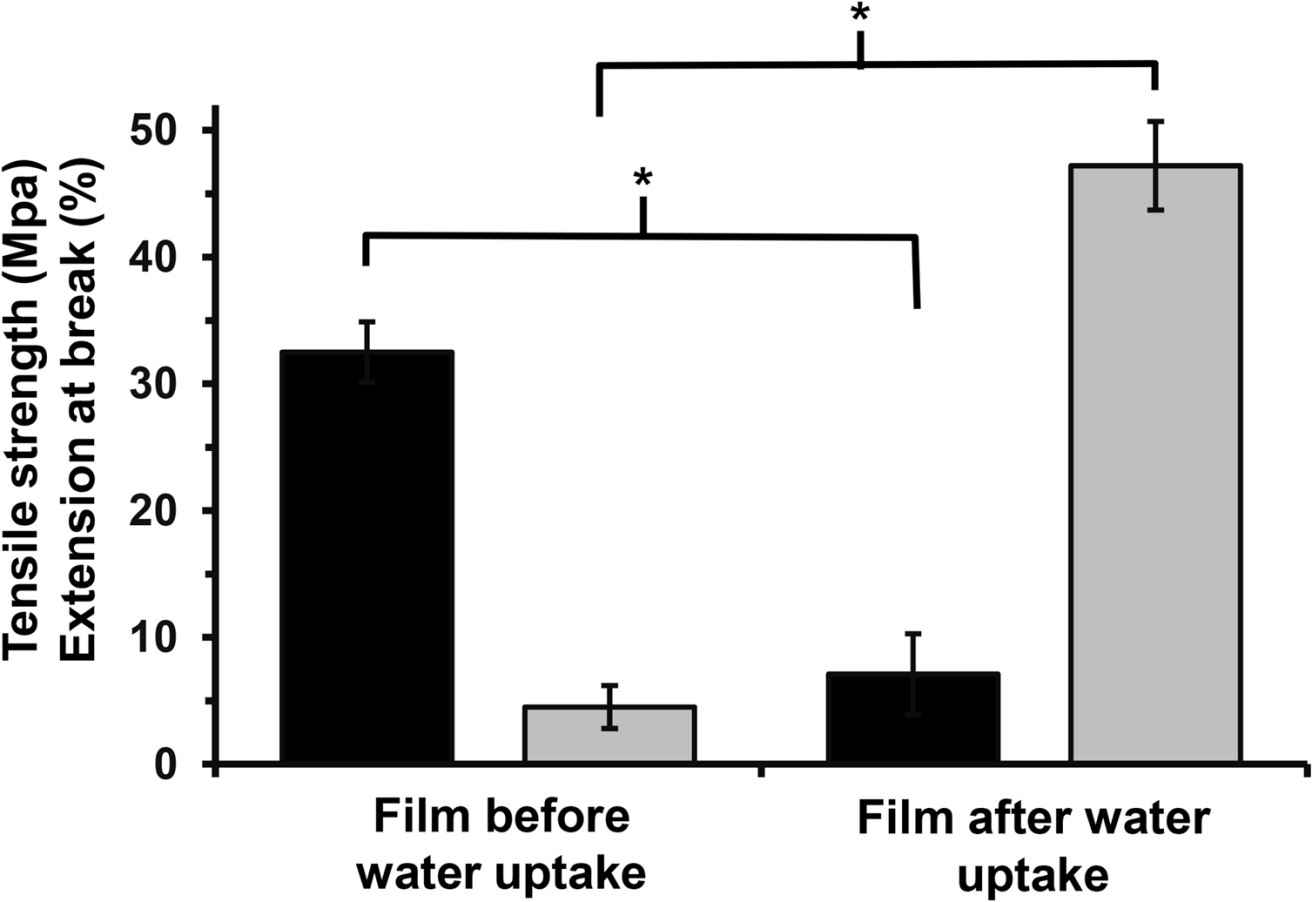
Variation in mechanical properties of solvent cast films before and after incubation in water (25°C, 2 h), fabricated from commercial chitosan with 85% DDA; (black square: tensile strength (MPa) and grey square: elongation at break (%), * Significant difference, P < 0.05, *n* = n ≥ 5).

The gelation qualities of chitosan are well established [35]. In this study, mechanical properties for chitosan films with a DDA of 85% were also assessed after incubating in sterile, saline buffer. This procedure resulted in a reversal of properties, with the hydrated chitosan films showing a significant reduction in tensile strength to 4.5 ± 1.7 MPa, while increasing the elongation at break to 47.2 ± 3.5% (Fig 5). This change in mechanical properties is consistent with swelling of the polymer films [11,34].

**Fig 6:**
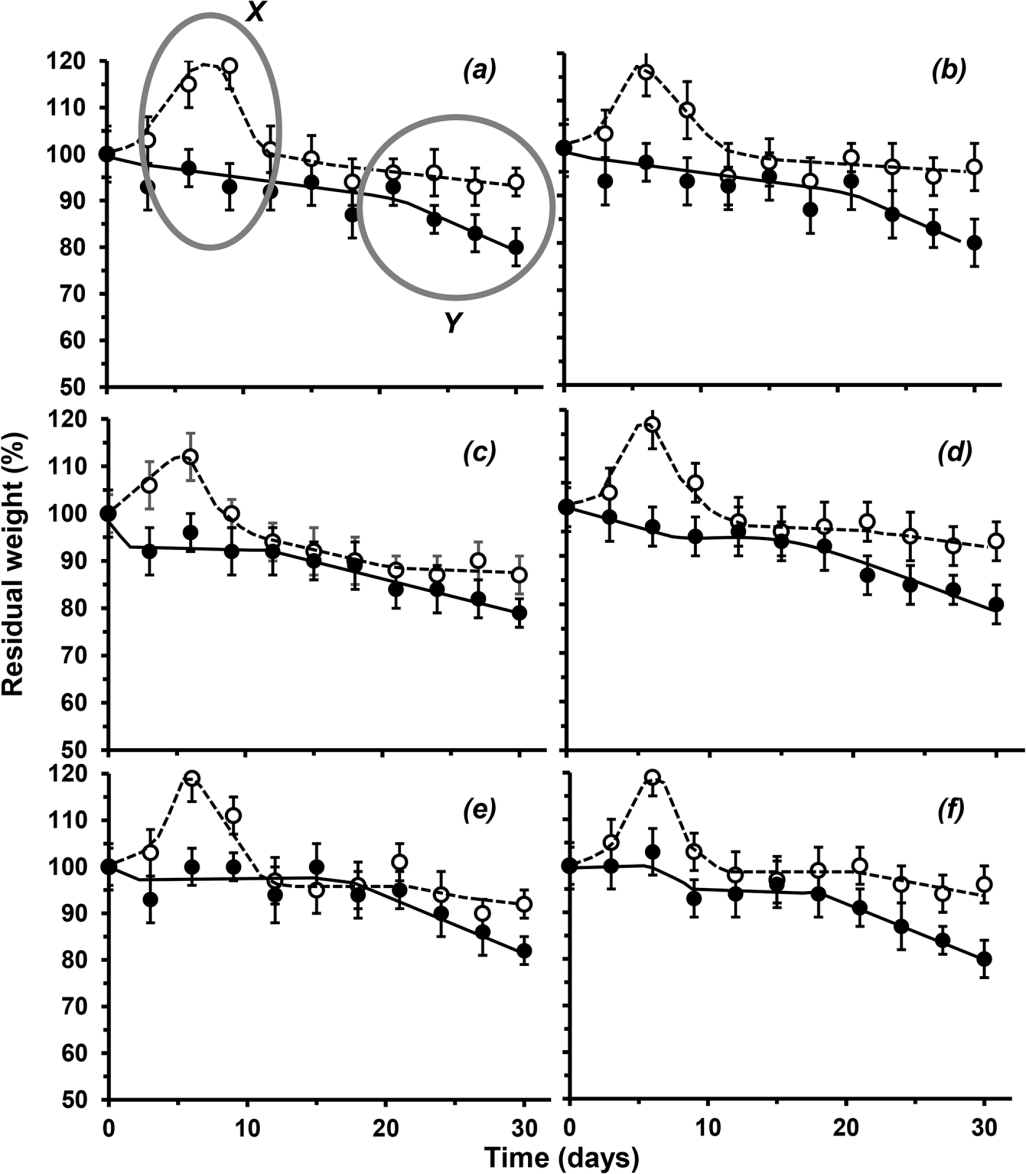
Variation in degradation for solvent cast films of different commercial chitosan samples with different DDAs, as measured by weight loss from original (%); *(a)* 72; *(b)* 73; *(c)* 75; *(d)* 77; *(e)* 79 and *(f)* 85% DDA; (37°C, pH 7.4, 120 rpm; white circle: abiotic degradation in PBS and black circle: biotic degradation in PBS with 2% w/v lysozyme).

When incubated in sterile physiological buffered saline at 37°C chitosan films initially absorbed buffer to show a significant peak increase to 112 ± 10% of the initial weight after 10 days, which is consistent with the swelling process [11]. Subsequent weight loss was roughly linear reaching a maximum (6%) after 30 days (Fig 6). Changes in crystallinity through DDA increase had no significant effect on susceptibility of the films to hydrolysis; chitosan films lost 6 ± 4% of their initial weight (P < 0.05). In contrast, biodegradation of the chitosan films using lysozyme showed no initial weight gain and, as anticipated, a greater degree of weight loss averaging at 80% of their initial weight after 30 days, consistent with Freire *et al* [36]. It can be speculated that initial penetration of the enzyme into the polymer matrix was responsible for chain cleavage preventing the hydration effects observed in its absence (Fig 6).

### Cellular response

**Fig 7:**
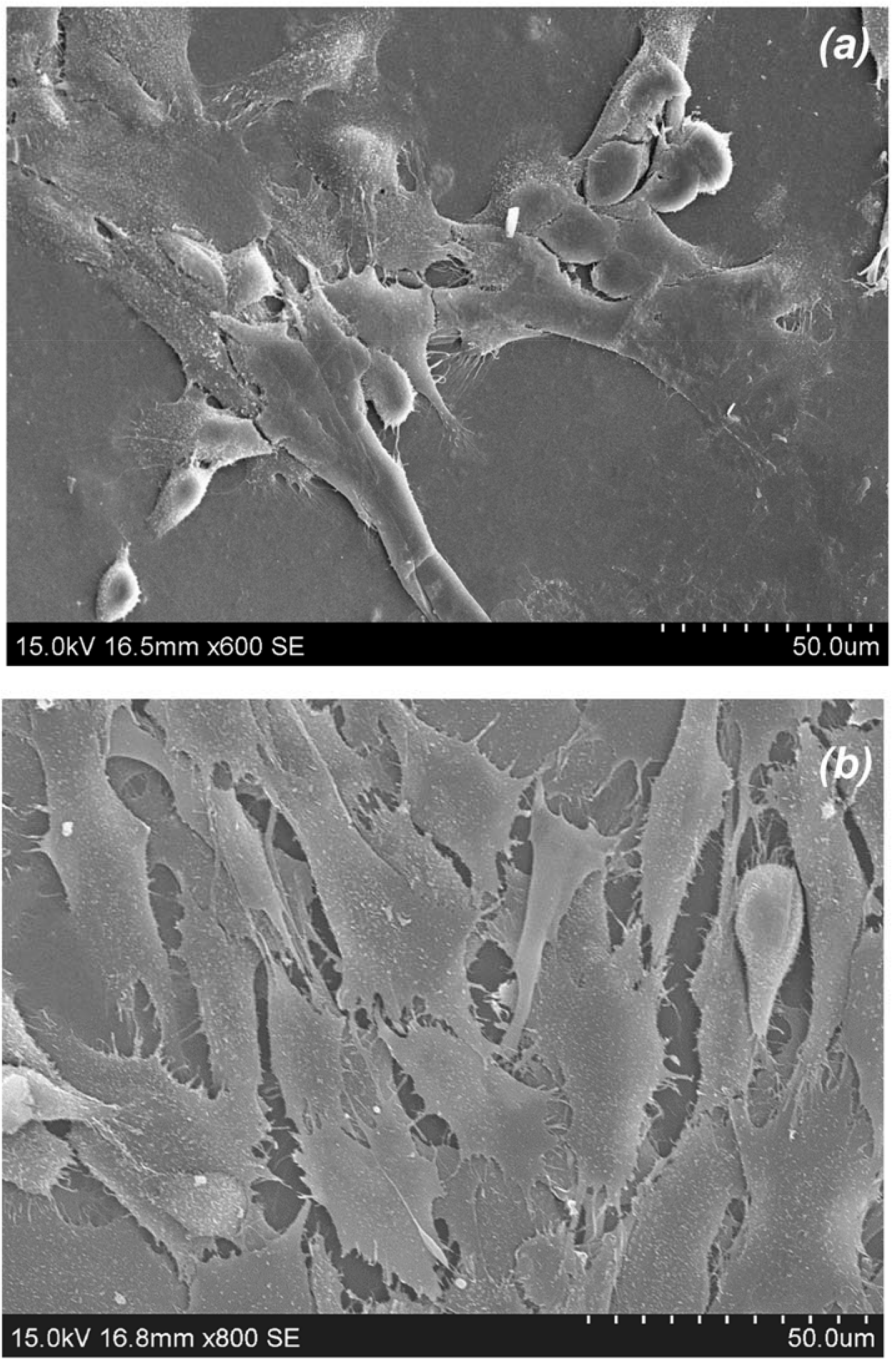
SEMs of olfactory ensheathing cells adhered to solvent cast films fabricated from chitosan with: *(a)* 72% DDA (Magn. x600) and *(b)* 85% DDA (Magn. x800).

**Fig 8:**
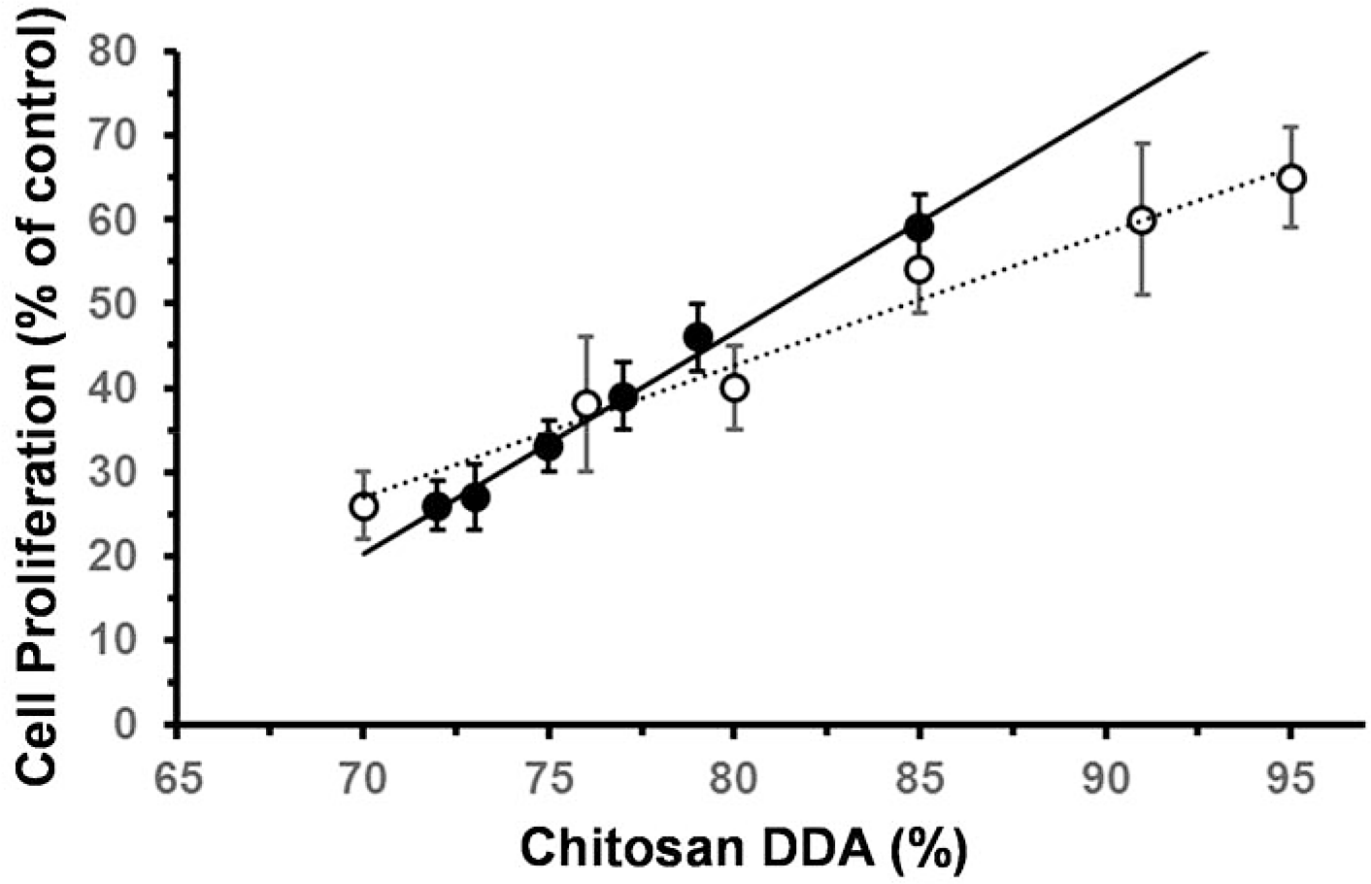
Variation in proliferation of olfactory ensheathing cells when cultivated (DMEM media, 5 days, 37°C, 5% CO_2_) on solvent cast films fabricated from commercial chitosan samples with different DDAs, expressed as % of asynchronous growth control; (black circle): this study and (white circle): Schwann cells as reported by [11]).

Olfactory epithelial cells (OECs) have been investigated for the repair spinal cord and peripheral nerve transection injuries [37–39]. OECs are a type of glial cell located in the olfactory bulb and epithelia, responsible for axon development in both the central and peripheral nervous systems, CNS & PNS respectively [38,40]. Foster and coworkers have recently shown that biomaterials can influence OEC migration by changing their protein regulation [28]. In the study here, OECs cultivated on chitosan films of 72% DDA showed a degree of spreading, but also a number of rounded blebs, suggesting some cytotoxicity (Fig 7A). In contrast, the same cell line simultaneously grown on chitosan films of 85% DDA showed much greater spreading with numerous filipodiae and no blebbing, suggesting better biocompatibility (Fig 7B). Cell proliferation after 5 days was consistent with the qualitative observations; increasing the DDA of chitosan films appeared to support an increase in OEC growth (Fig 8) [11]. A linear trend of increasing OEC proliferation relative to cells in asynchronous growth with DDA increase (R^2^ = 0.9918) is consistent with the growth of Schwann cells as reported by Wenling *et al*. (R^2^ = 0.9782) [11]. In the study here, biocompatibility was further examined through cell health.

**Fig 9:**
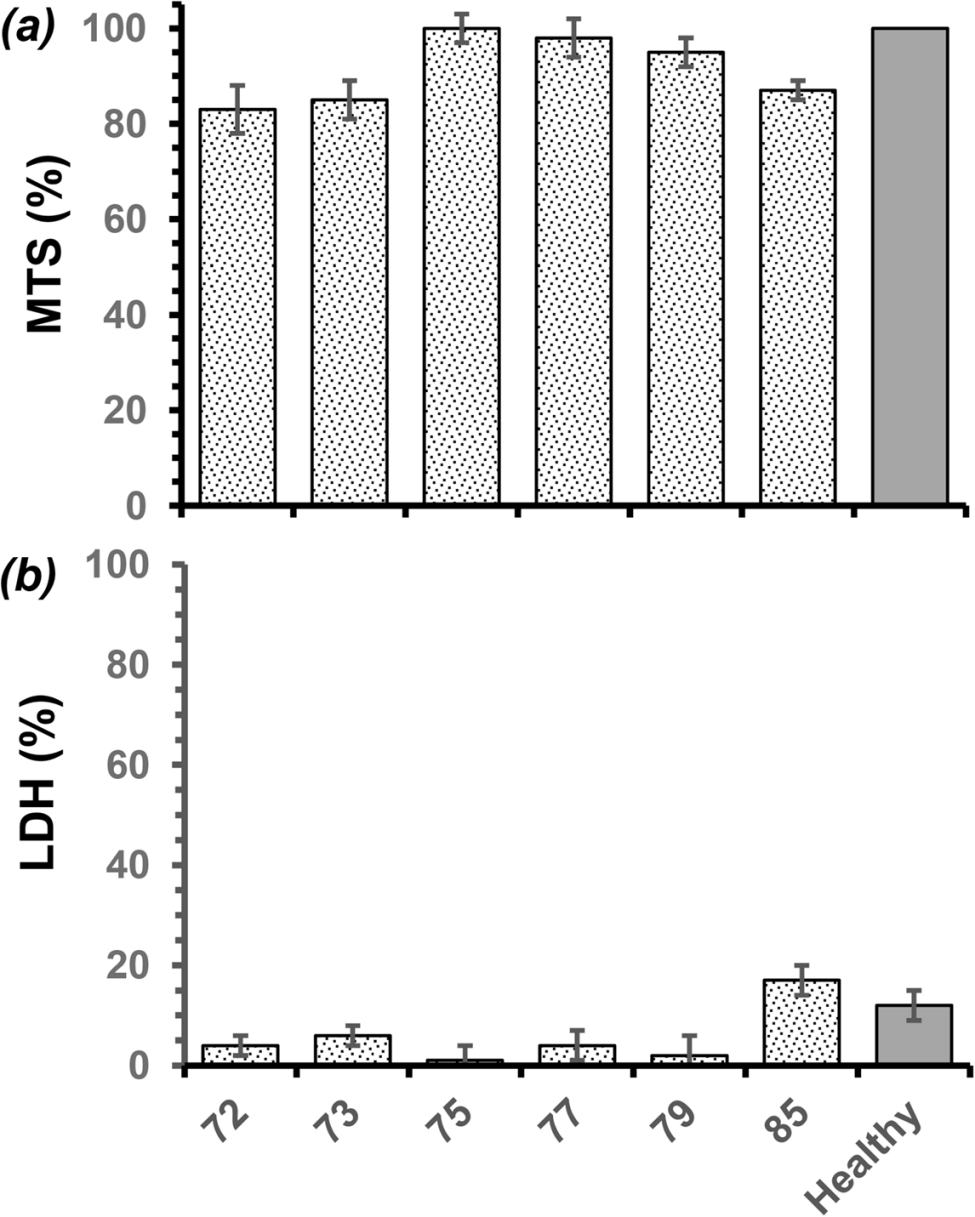
Variation in health of olfactory ensheathing cells when cultivated (DMEM media, 5 days, 37°C, 5% CO_2_) on solvent cast films fabricated from commercial chitosan samples with different DDAs, as measured by: *(a)* mitochondrial activity, expressed as % of cells in asynchronous growth (100%), and membrane integrity expressed as % of lysed cells (100%); (* Significant difference, P < 0.05, n ≥ 3).

**Fig 10:**
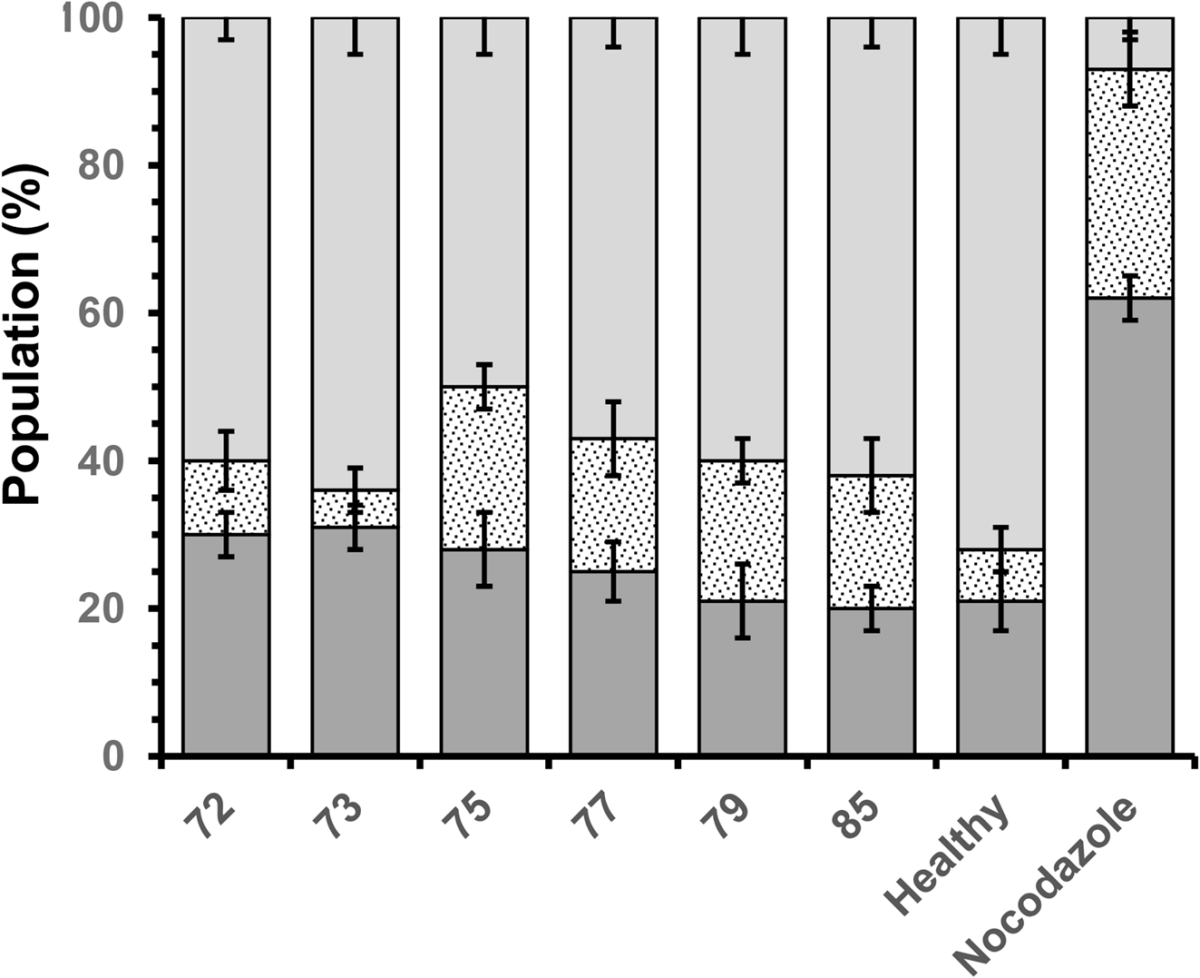
Variation in health of olfactory ensheathing cells when cultivated (DMEM media, 5 days, 37°C, 5% CO_2_) on solvent cast films fabricated from commercial chitosan samples with different DDAs, as measured by apoptotic indices; (light grey square) live; (spotted square) early apoptotic and (dark grey square) necrotic.

Consistent with the proliferation data and cell observations, OECs cultivated on chitosan films with DDAs of 72 and 73% showed significantly less mitochondrial activity, as measured by MTS, around 84 ± 4% of that of cells exhibiting asynchronous growth in the absence of biomaterials (assigned 100%, Fig 9A). While cells cultivated on films fabricated from chitosan with 75 to 79% DDA showed no significant difference from the asynchronous OECs, those cultivated on films with 85% DDA possessed 87 ± 2% of the mitochondrial activity exhibited by the asynchronous cells (Fig 9A). The data suggests that OECs proliferating on the films of chitosan films with comparatively high DDA (85%) had reached over confluence. Similarly, there were no significant differences in membrane permeability, (Fig 9B). Cells grown in the presence of nocodazole as a positive control had a significant percentage of their population, 62 ± 3%, necrotic and 31 ±7% apoptotic. In contrast, the control of cells exhibiting asynchronous growth possessed 21 and 7% of the cell populations necrotic and apoptotic respectively, validating the experiment (Fig 10). Consistent with the cell proliferation data, OECs cultivated on chitosan films with increasing DDA showed less necrotic cells. On films with a chitosan DDA of 72%, 30 ± 3% of the population were necrotic, compared to 20 ± 3% of their counterparts on chitosan with 85% DDA. However, populations grown on chitosan films with 79 and 85% DDA appeared to possess greater proportions of cells exhibiting early apoptosis, 18 ± 4% compared to 10 ± 3% for those grown on chitosan with 72% DDA (Fig 10). Thus, OECs cultivated on films fabricated from chitosan with 79 and 85% DDA exhibited similar necrotic cell percentages as cells exhibiting asynchronous growth but greater proportions of the population exhibiting apoptosis, approximately 21% (Fig 10).

**Fig 11:**
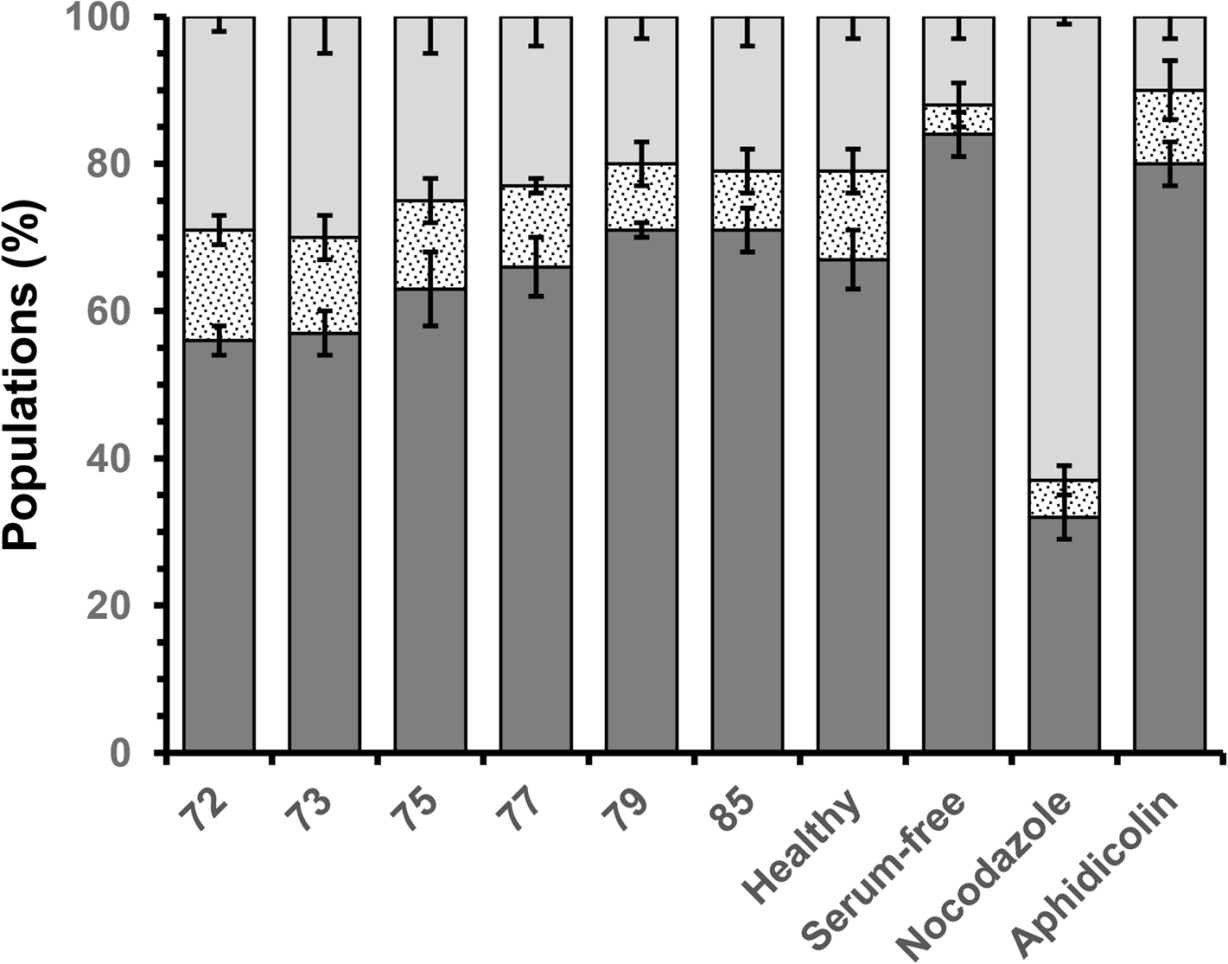
Variation in health of olfactory ensheathing cells when cultivated (DMEM media, 5 days, 37°C, 5% CO_2_) on solvent cast films fabricated from commercial chitosan samples with different DDAs, as measured by cell cycle progression; cell populations in the G0/G1 phase (dark grey square); the S phase (spotted square) and the G2/M phase (light grey square).

A more detailed examination of OEC response to the chitosan films was determined through cell cycle analysis, measuring the proportion of cells within each of the phases ‘G_0_/G_1_’ (senescence/rest), ‘S’ (DNA replication) and ‘G_2_/M’ (DNA repair/cell division) [41]. Aphidicolin is a mycotoxin that inhibits the activity of DNA polymerase a, arresting cells in the G_0_/G_1_ phase, while nocodazole is antimicotic agent that depolymerises ECM microtubules, arresting cells in the G_2_/M phase, preventing cell division. In the study here, OECs cultivated in the presence of aphidicolin and nocodazole showed 80 ± 3 and 63 ± 1% of their populations arrested in the G_0_/G_1_ and G_2_/M phases respectively, validating the experiment (Fig 11). OECs cultivated on chitosan films with comparatively low DDAs (72 and 73%) revealed significantly less cells in the G_0_/G_1_ phase, approximately 57 ± 3%, when compared to their counterparts cultured on films with comparatively high DDAs (79 and 85%), approximately 71 ± 2% (P < 0.05, Fig 11). Thus, chitosan films with 72 and 73% DDA appeared to stimulate OEC cell cycling with greater proportions of cells in the S and G_2_/M phases (14 ± 3 and 30 ± 3% respectively) than their counterparts on films with 79 and 85% DDA (8 ± 2 and 20 ± 3% respectively). However, the cycle distributions of OEC populations on chitosan films with 79 and 85% showed no significant difference from OEC population exhibiting asynchronous growth. In comparison with the apoptotic indices and qualitative observations, the cell cycle analysis suggests that chitosan films with comparatively lower DDAs, while cytocompatible induced cellular stress. In contrast, chitosan films with comparatively higher DDAs show good compatibility with measures of OEC health similar to those of cells in asynchronous growth.

### Surface properties

**Fig 12:**
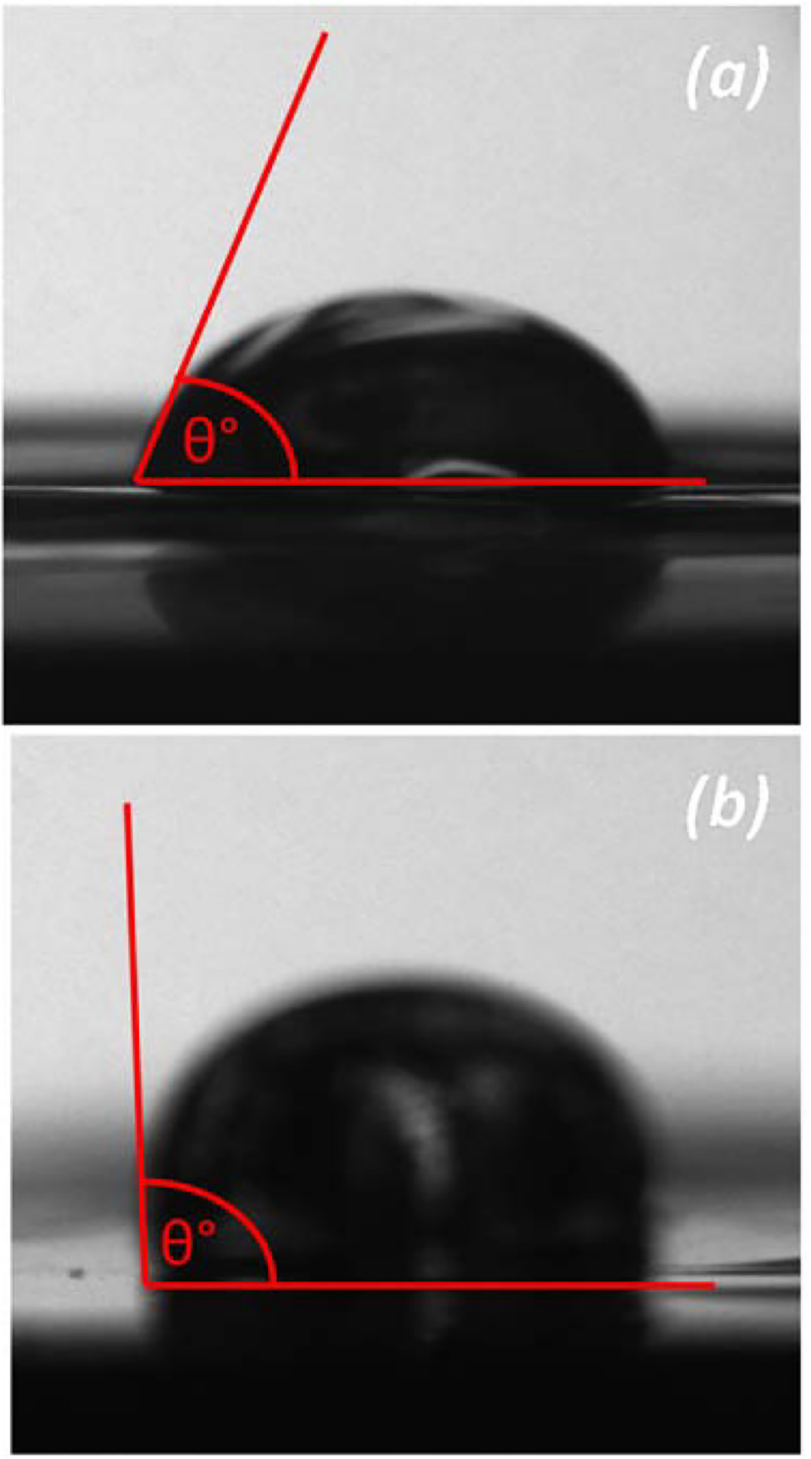
Images showing change in surface hydrophobicity for solvent cast films fabricated from commercial chitosan samples with *(a)* 72 and *(b)* 85% DDA.

**Fig 13:**
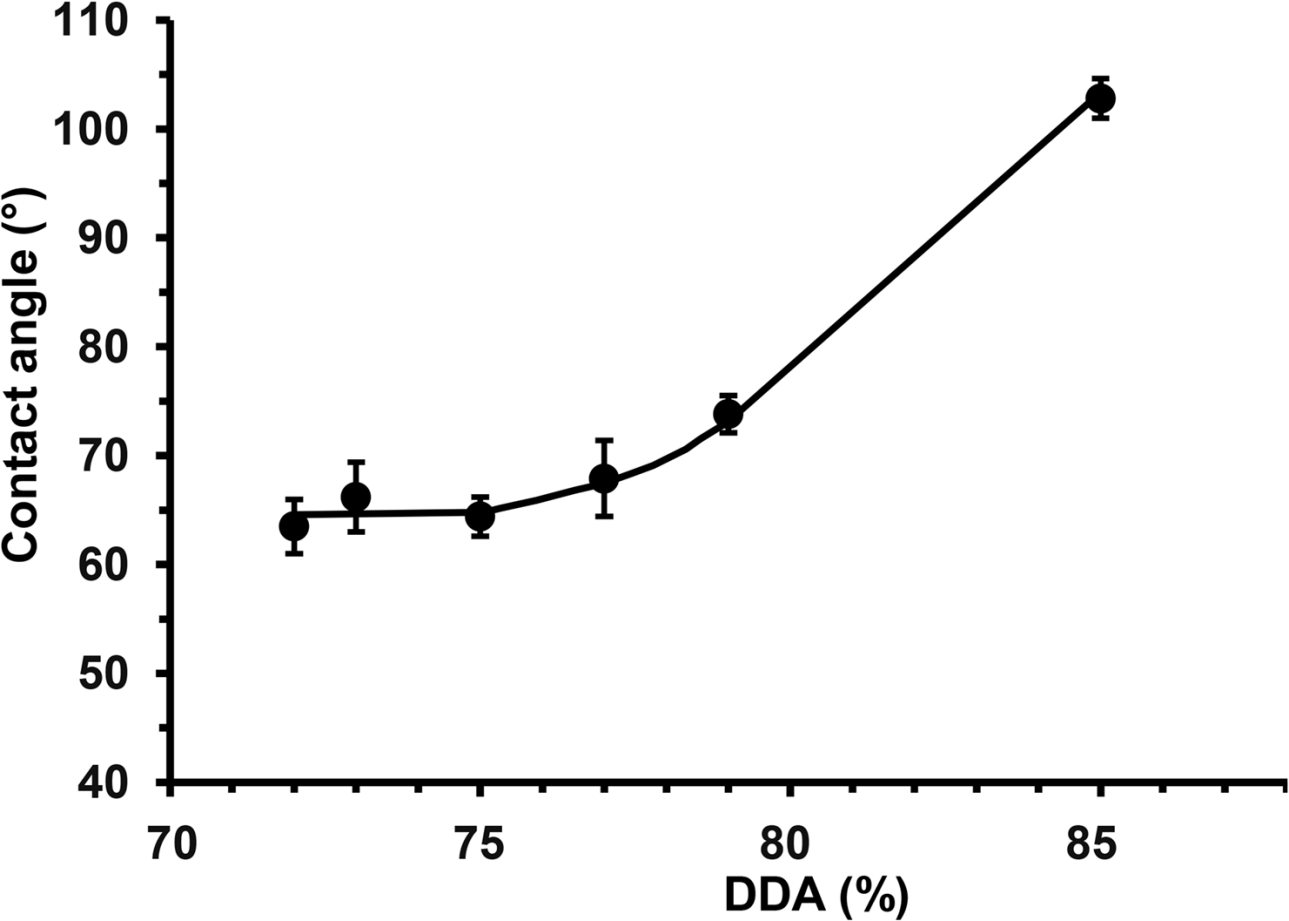
Change in hydrophobicity of solvent cast films fabricated from commercial chitosan samples with different DDAs, *(a)* surface hydrophobicity as measured by water contact angle and *(b)* bulk hydrophobicity as measured by water uptake, (* Significant difference, P < 0.05, *n* ≥ 10).

Surface chemistry and morphology are known to influence cellular response [42,43]. In the study here, Fig 12 clearly shows that the chitosan film with a DDA of 73% had a significantly lower water contact angle than its counterpart with a DDA of 85%. Films with DDAs of 72 to 75% had similar contact angle of about 65 ± 3°, increasing DDA further resulted in a gradual increase in contact angle to 103 ± 2° (Fig 13). These results suggest a gradual increase in hydrophobicity as DDA increases. However, Tomihata *et al*. report that increasing chitosan DDA decreases hydrophobicity, while Wenling *et al*. claims that there is no pattern to the relationship between DDA and hydrophobicity [44,11]. These contradicting reports can be readily explained by differences in chitosan film preparation and their effects on the surface charge. Changes in availability of the amine groups may not only have affected water contact angles, but adhesion of biological macromolecules when incubated in culture media, thus supporting the OEC proliferation trend observed in Fig 8.

**Fig 14:**
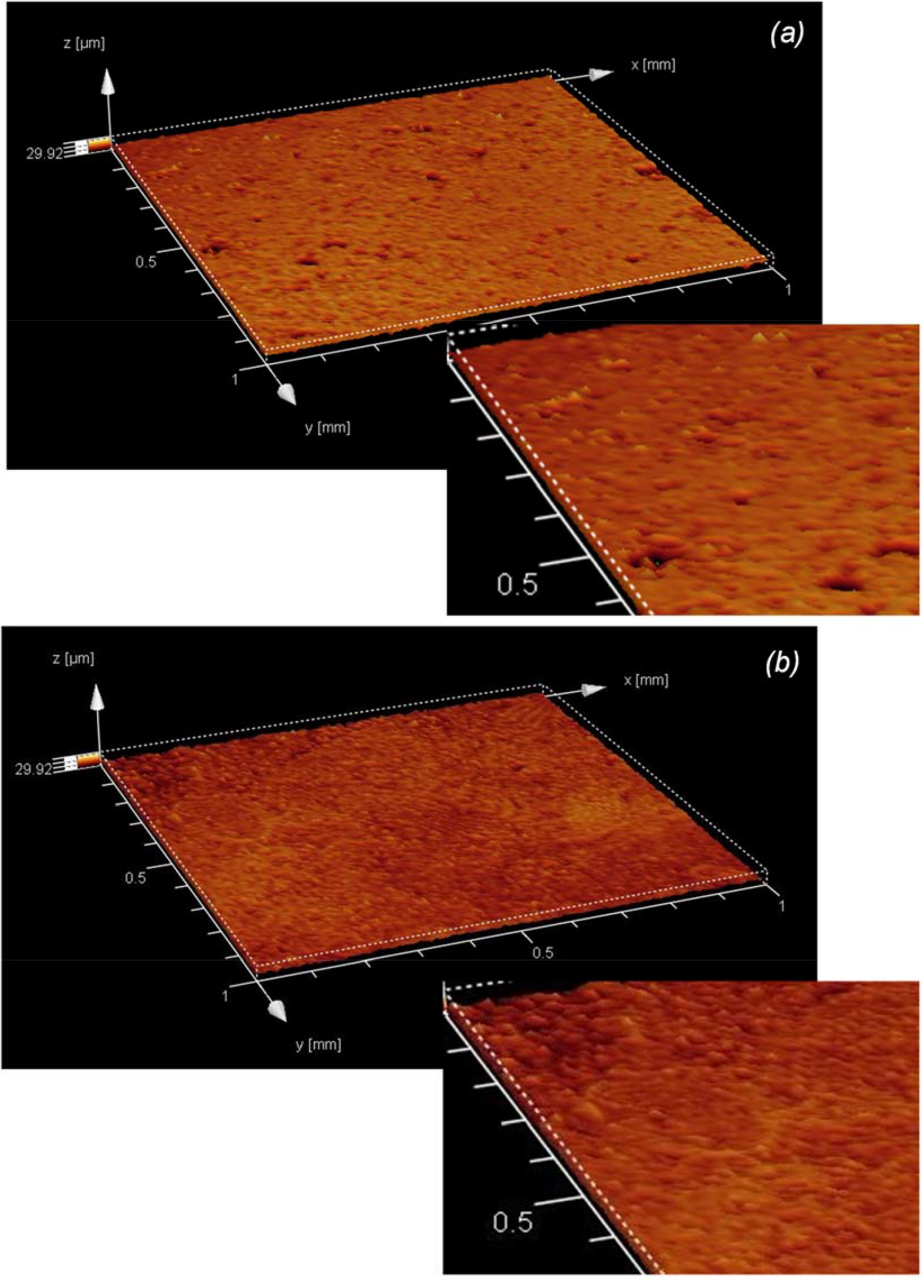
CLSM images illustrating the microtopographies of solvent cast films fabricated from commercial chitosan samples with a DDA of *(a)* 72 and *(b)* 85%.

**Fig 15:**
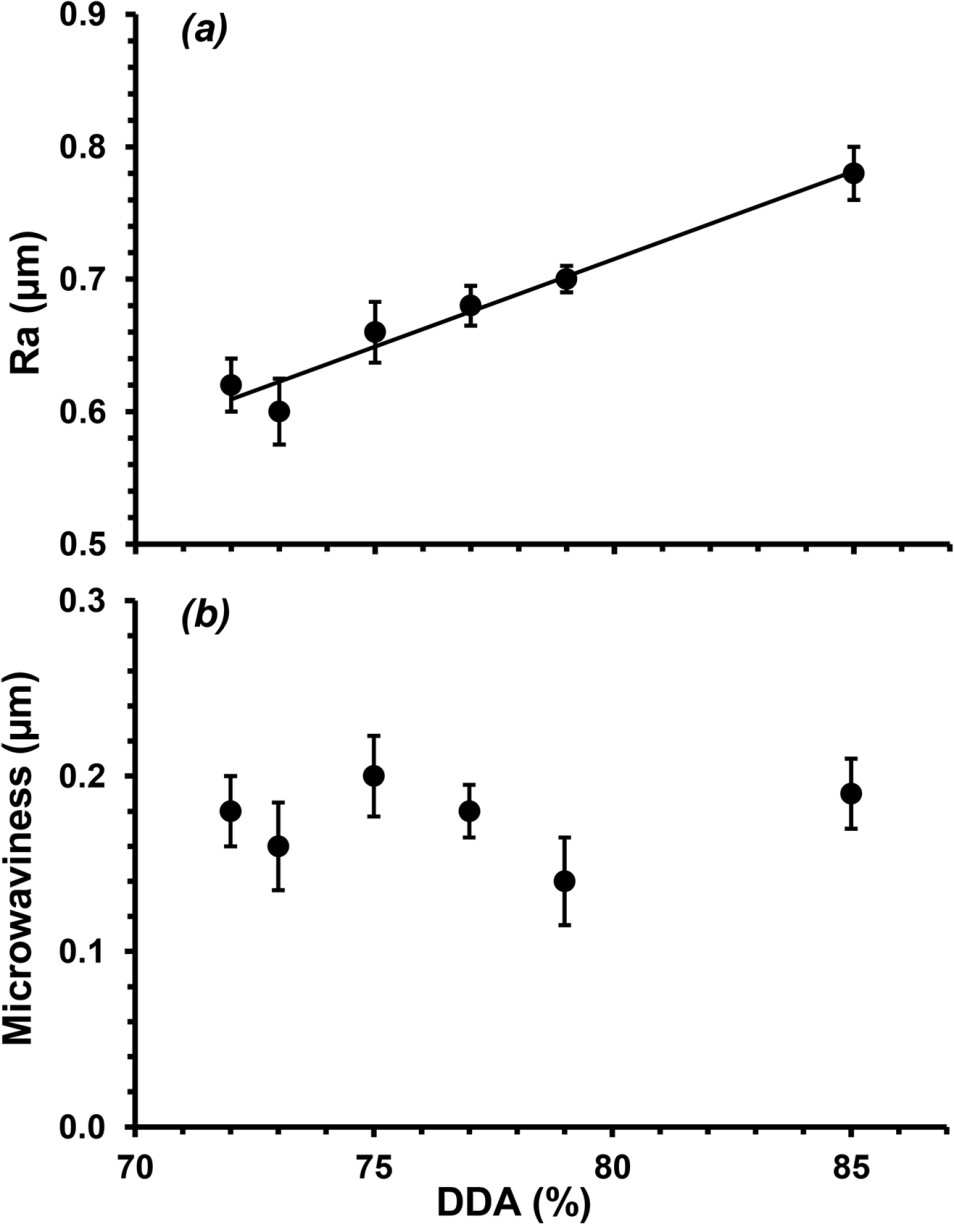
Variation in surface microtopograpies of solvent cast films fabricated from commercial chitosan samples with different DDAs as determined by *(a)* average surface roughness (Ra) and *(b)* microwaviness.

Water contact angle measurements, as well as the adhesion and proliferation of both mammalian and microbial cells, are also influenced by surface topography [42,43,21]. Fig 14A shows the microtopography of a film fabrication from chitosan with a DDA of 72% as determined through confocal laser scanning microscopy; little difference can be discerned when compared to a film fabricated from chitosan with a DDA of 85% (Fig 14B). However, quantitative analysis of these depth maps showed significant differences in their average surface roughness, R_a_. There was a linear increase in R_a_ with increasing DDA, from 0.62 ± 0.02 to 0.78 ± 0.02 μm for films fabricated from chitosan with 72 and 85% DDA respectively (Fig 15A). Chung *et al*. has reported that increases in surface roughness even at 10^1–^10^2^ nm scales can enhance cell adhesion to polymer surfaces [43]. Thus, the trend in surface roughness determined here is consistent with the trend for cell proliferation, suggesting that the rougher surface also supported cell adhesion and proliferation. In contrast, the harmonic component of these films, ‘microwaviness’, showed no significant difference with DDA variation, suggesting no regular arrangement to the microtopographies (P < 0.05, Fig 14B). Furthermore, Clasen *et al*. has suggested that roughness and porosity can decrease tensile strength due to less stability in the molecular structure [45].

## Conclusions

Chitosan is an attractive biomaterial with a range of current and potential biomedical applications. Manipulation of chitosan DDA to achieve specific properties appears feasible. However studies investigating the influence of DDA on the properties of chitosan are often contradictory. The study here demonstrates the variation in physiochemical, mechanical and biological properties for a range of commercial chitosans within a narrow DDA range (72–85%). Standard solvent cast films show patterns in mechanical properties that appear to be a function of both crystallinity and gelation. In contrast, cell proliferation and health were most probably influenced by significant changes in surface hydrophobicity and microtopography. Finally, perceived patterns in property changes are subject to change based on potential variations in DDA analysis. NMR examination of the chitosan samples here revealed significant differences depending upon which peaks were selected for integration. Furthermore, the differences between DDA values in our NMR examination and those reported by the commercial suppliers were significant and this may also be a source of concern when selecting commercial chitosans for biomaterial research.
